# Utility of urinary sodium to guide diuresis in acute decompensated heart failure

**DOI:** 10.1093/eschf/xvag142

**Published:** 2026-06-22

**Authors:** Antonia Salem

**Affiliations:** ESC Heart Failure

**Keywords:** Urinary sodium, Acute heart failure, Diuretic therapy, Clinical practice

I read with great interest the national survey by Cobo Marcos et al. examining the utilization of urinary sodium (UNa) guided diuretic therapy in acute decompensated heart failure (ADHF) across Spain.^1^ This report addresses an important and understudied gap: the persistent discrepancies between guideline recommendations and bedside practice. Traditional measurements of diuretic response, such as daily weights and urine output measurements—can be inconsistent and logistically burdensome. UNa offers a more objective, practical alternative. The breadth of the survey across four specialties provides a valuable picture of current clinical behaviour.

The report finds that only 18% of participating physicians use UNa routinely, despite 78% acknowledging its clinical utility, highlighting an important evidence-practice gap. I draw particular attention to the emergency physicians; among whom 42% reported no use and 33% were unable to identify an appropriate resistance threshold—the highest rate of diagnostic uncertainty across all specialties. This is of specific concern, as European Society of Cardiology (ESC) guidelines state UNa monitoring is most accurate within the first 24–48 hours.^2^ As emergency physicians are typically the first to assess patients, they are often responsible for prescribing the first dose of diuretic. Targeted education campaigns should therefore prioritize this group.

The near absence of acetazolamide, a carbonic anhydrase inhibitor, as a therapeutic response (3%) deserves closer examination. The authors attribute this to the limited awareness of its efficacy, citing the ADVOR trial.^3^ I propose that cost and regulatory context are additional barriers. In the United Kingdom, acetazolamide has not yet been licensed to treat ADHF despite the strength of available evidence. Notably, it is significantly higher in cost than furosemide, one of the more commoner diuretics therapies. A formal cost-effectiveness evaluation and integration into prescribing guidelines are prerequisites to meaningful uptake in many healthcare systems.

I would also highlight a notable omission from the survey’s included specialties. Given that heart failure is significantly more common in our ageing populations, elderly care departments carry a substantial burden of ADHF management. Their inclusion in the survey would have been meaningful. In addition, the offer of fixed options of <50, <70, or <100 mmol/l may have invertedly guided responses in those with partial knowledge.

To contextualize these findings for a UK setting, I present preliminary data from an ongoing audit at our institution of 48 consecutive patients admitted with ADHF over a two-month period. We assessed whether a UNa sample was obtained two hours after the first diuretic dose, and whether the result was documented and used to guide titration, in line with ESC guidelines. Early findings show uptake is below 20% across all three criteria, consistent with, yet poorer than, use reported by Cobo Marcos et al. The primary barrier identified is poor awareness of recent guidelines and UNa’s clinical utility.

The ENACT-HF trial demonstrated that a protocolized UNa guided strategy was associated with a 64% increase in natriuresis within 24 hours and a shorter inpatient stay.^4^ Given that unplanned hospital admissions represent one of the highest costs to healthcare systems globally, tools that reliably reduce length of stay warrant urgent adoption. The PUSH-AHG trial, however, found no significant reduction in 180-day rehospitalization, suggesting that whilst UNa guided diuresis optimizes short-term decongestion, the long-term trajectory of heart failure may remain the same.^5^

Finally, I note that the 2025 ESC expert consensus document, published after the survey was conducted, now recommends a UNa threshold of >70 mmol/l as an indication of inadequate diuretic response.^2^ This represents a meaningful clarification over the previously cited range of 50–70 mmol/l, which itself reflected the two most selected survey options. A single, unambiguous value should reduce the inter-clinician variability in threshold interpretation that this survey has exposed; future surveys should incorporate the updated threshold to enable meaningful longitudinal comparison.

Current evidence base, combined with this report, concludes that UNa guided diuretic therapy is clinically effective. Inadequate measures of diuresis cause delays in necessary dose escalation or initiation of combination therapy; this drives diuretic resistance, increases length of stay in hospital, and is associated with poor patient outcomes.^2^ The remaining barriers to increasing the use of UNa as a measure of diuresis are educational and implementational, rather than scientific, and are therefore addressable.

## Declarations

### Data Availability

No data were generated or analysed for this manuscript.
